# Successful Retention Strategies for Nurses in Home Visiting Nursing Services: A Scoping Review

**DOI:** 10.1111/jan.70020

**Published:** 2025-06-16

**Authors:** Vari M. Drennan, Joel Coll Ferrer, Claire Thurgate, Lihua Wu, Mary Halter, Erkan Alkan

**Affiliations:** ^1^ Institute of Health, Education and Science Kingston University London UK; ^2^ School of Nursing, Allied and Public Health Kingston University London UK

**Keywords:** district nursing, home health care nursing, retention, scoping review, visiting nursing, workforce

## Abstract

**Aim:**

To identify successful strategies and underpinning mechanisms for retaining nurses in home visiting nursing services.

**Design:**

Scoping review.

**Data Sources:**

MEDLINE, CINAHL, Google Scholar, Theses Global Databases (1 January 2000 to 23 November 2023); international nursing organisations websites (January–April 2024).

**Review Methods:**

The methods followed the Joanna Briggs Institute guidance. Two researchers independently screened and reviewed, with disagreements resolved through discussion. Included papers were analysed for underlying mechanisms.

**Results:**

Of 1219 records identified, seven met the criteria. Four papers reported senior administrators' experience of successful multiple types of strategies (unspecified), but none reported retention outcomes. Three papers reported evaluations of initiatives providing clinical and peer support to nurses new to home visiting nursing. All three papers reported improved retention rates at 12 months in comparison to the year previous, although there was no consideration of other potentially influencing factors. We identified eight underlying mechanisms in the seven papers: (1) finance incentives; (2) work schedule flexibility for individuals; (3) team level management; (4) positive feedback on job performance; (5) team level interpersonal relationships; (6) the work organisation and resources; (7) support to individual's development in knowledge, clinical skill and confidence and (8) participation in organisation's decision making.

**Conclusion:**

This review identified noticeable few papers over a time when all countries have been trying to address the growing health needs of the older populations. The gap in evidence as to the most effective combinations of retention strategies for home visiting nursing requires urgent attention. Clinical leaders and managers require evidence to inform their strategies for retaining home visiting nurses in order to provide high quality care as more health care systems increase the provision of acute, chronic, and palliative care in patients' own homes.

**Reporting Method:**

This paper conforms to PRISMA reporting guidelines for scoping reviews.

**Patient or Public Contribution:**

No patient or public contribution.

Summary

- What problem did the study address?
  ○ Retaining nurses in home visiting nursing services (also known as home healthcare, community or district nurses in some countries) is reported to be problematic in many countries. Nursing in patients' homes is a very different work environment from hospitals. This review addressed the problem of which strategies are most successful in retaining nurses in home visiting nursing services.
- What were the main findings?
  ○ There were only seven papers published over 24 years which addressed this problem. Managers and agencies report employing multiple types of strategies to achieve success in retention. However, there is no published evidence of the impact of different types of retention or combinations of retention strategies for nurses in home visiting nursing services. The evidence from clinical and peer support programmes for new to home visiting nursing services shows promise in retention.
- Where and on whom will the research have an impact?
  ○ This study will be of value to nurses leading home visiting nursing services and teams. It will provide information of the range of mechanisms involved in successful retention strategies for all nurses as well as evidence of the promise of targeted clinical and peer support for those new to nursing in patients' homes.
- What does this paper contribute to the wider global clinical community?
  ○ The review identifies a paucity of evidence to guide nurses and nurse managers where best to focus their efforts in retention strategies for nurses in home visiting nursing services.
  ○ The review identifies eight underlying mechanisms in retention strategies that require further testing within communities of nurses and nurse managers.
  ○ The review provides further evidence of the potential value of tailored induction and support programmes for nurses new to working in people's homes.

## 1 Background

Internationally, health services have had workforce challenges before and since the COVID‐19 pandemic (Agyeman‐Manu et al. 2023). All countries are facing increased population health needs, increased patient demand at the same time as recovering from the impact of the pandemic. The policy responses to these problems in many countries have been to increase health services outside of hospitals and delivered in people's homes, such as hospital at home (World Health Organisation 2023; Levine et al. 2021). These plans are dependent on having the appropriately skilled and available nurses.

Many, but not all, countries have home visiting nursing services which are variously known as community nursing, home health care, home visiting, visiting nurses, district nursing (Drennan 2019). Home visiting nursing services predominantly provide nursing care and treatment to housebound, older adults with multiple chronic conditions, as well as palliative and end of life care (Drennan 2019). There is international variation in the organisation and educational requirements of nurses in home visiting nursing services. Home visiting nursing services are provided by organisations which can be for‐profit, not‐for‐profit, non‐governmental or state run health services (Drennan 2019). The funding sources also vary between countries and can be from health insurance, government funding, or direct patient payments (Drennan 2019). In most countries, the registered nurses (RNs) work in teams which can include practical nurses (if they exist in that country) and non‐professionally qualified assistants (Drennan 2019). In a few countries, there are specific post qualifying district nurse education programmes, but not all RNs are required to have it (Drennan 2019). Prior and since the pandemic there have been reported problems with high vacancy and turnover rates of nurses in district nursing services in countries such as the United States (USA) and Sweden (Labour Market Tendency Survey 2 2020; Gaines 2021). This study addressed questions as to the evidence for effective strategies in retaining nurses in home visiting nursing services.

The World Health Organisation (WHO) and the International Council of Nurses (ICN) have argued that nursing services must have retention strategies to address growing population needs and global nursing shortages (World Health Organisation 2016; Buchan et al. 2022). Neither the WHO nor the ICN retention guidance documents referred specifically to home visiting nursing. However, to nurse in people's homes is a very different work environment from nursing in hospitals or other care facilities. For example, nurses in home visiting nursing services usually work alone both in travelling to and in visiting their patients in their homes. They are consequently at some distance from colleagues when having to make clinical and professional decisions, often in less‐than‐ideal situations (Drennan et al. 2025). A Canadian study reported nurses in home visiting nursing services as having significantly greater concerns over isolation from their agency base, personal safety issues and costs of maintaining a car for work than did public health nurses and those working in clinics (Armstrong‐Stassen and Cameron 2005). This evidence suggests that effective retention strategies for nurses in home visiting nursing may need to differ from those for nurses working in other types of services.

Our preliminary investigations for reviews of retention strategies for nurses in home visiting nursing included searching PubMed, PROSPERO and Google Scholar databases in October 2023. Five reviews were identified with ambiguous titles and abstracts (Hariyati and Nurdiana 2018; Twigg and McCullough 2014; Lartey et al. 2014; Williamson et al. 2022; Brook et al. 2019). However, a detailed reading of these reviews found they only reported strategies for nurses employed in hospital or other health care facilities. We found one review of evidence concerning the retention of district nurses (Chamanga et al. 2020). This review only included studies of nurses' reported intention to leave their jobs in home visiting district nursing and did not report on the effectiveness of any retention strategies (Chamanga et al. 2020).

Given the absence of reviews on effective retention strategies for district nursing services, we wanted to investigate: the types of available evidence, how research had been conducted in this field and what the gaps were in the knowledge base. Our purpose was to provide a synthesis of evidence of value to clinical managers and policy makers for home visiting nursing services. Our intentions most closely aligned to the purposes for undertaking a scoping review (Munn et al. 2018). Our scoping review was further underpinned by our knowledge that nurses within these services are not a homogeneous group either terms of the stage of their nursing careers or in their educational preparation for nursing in people's homes (Drennan 2019). Consequently, we planned to employ analytical techniques from realist review methods to our findings (Pawson et al. 2005). The methods of realist reviews have been designed to explain the success or failure of social intervention policies (Pawson et al. 2005). Realist reviews draw on philosophical underpinnings with applications in policy evaluations (Pawson et al. 2005). In essence they seek to answer what works, for who and in what circumstances, by identifying relationships between contexts, mechanisms and outcomes and offering explanatory theories (Pawson et al. 2005).

The aim of this review was to explore the range, effectiveness and underlying mechanisms of retention strategies used specific to home visiting nursing services.

The research questions were:

1. What are the range of strategies employed by organisations to retain nurses in home visiting nursing services?
2. What are the explanatory mechanisms that underpin the strategies?
3. What is the evidence of the impact and outcome of the strategies?

## 2 Methods

The method followed the Johanna Briggs Institute guidance on scoping reviews to ensure a systematic and transparent process (Peters et al. 2020). The review protocol was published prior to commencement in 2023 (Drennan et al. 2023). We report here using the PRISMA guidance for reporting scoping reviews (Tricco et al. 2018).

## 3 Eligibility Criteria

Literature eligibility criteria were clarified through the use of the population, concept and context (PCC) framework (Peters et al. 2020):

- *Population*: nurses in home visiting nursing services and the organisations employing the nurses (and all the permutations of names for those nurses e.g., home visiting, community).
- *Concept*: literature that describes strategies or policies in use which were designed to retain nurses in home visiting nursing services in their jobs and organisations; and describes any outcomes from those strategies.
- *Context*: high‐ and middle‐income country settings, as defined by the World Bank (The World Bank 2024), in which organisations employ nurses in home visiting nursing services.

The inclusion and exclusion criteria were developed from the PCC framework (Table 1). The start year of the review was 2000 as this was the year the global shortages of nurses were first identified and subsequently described as a global crisis (World Health Organisation 2016). The inclusion of only similar income countries was to ensure any evidence found was broadly comparable within contextual resources for health services (The World Bank 2024).

**TABLE 1:** Paper inclusion and exclusion criteria.

	Inclusion criteria	Exclusion criteria
Population	Nurses in district nursing services for adults and the organisations employing the nurses (and all the permutations of names for those nurses e.g., home visiting, community)	Nurses working in other settings for example, clinics, care homes, hospitals Nurses providing public health/health promotion activities only Nurses working only with children and their families Non‐professionally qualified staff working with the nursing team e.g., health care assistants, home care aides Home care aides and workers outside of nursing services
Concept	Literature that describes strategies or policies in use which were designed to retain home visiting community nurses in their jobs and organisations; and describes any outcomes from those strategies	Literature that describes recruitment or training strategies without reference to retention Literature that describes surveys of home visiting/community nurses investigating job satisfaction, intentions to leave, mental and emotional health (including burnout), including those that investigate mediating factors on intentions to leave Literature that describes an individual's opinion of possible strategies, rather than implemented strategies
Context	High‐ upper middle‐ and lower middle‐income countries as defined by the World Bank (Smith‐Stoner and Markley 2007)	Low‐income countries. The split of countries according to income was used to find a homogeneous sample of studies with similar levels of health systems and health resources
Date	January 2000 onwards	Prior to January 2000
Language	English	Non‐English papers
Type of publication	Peer reviewed research studies of any type, non‐peer reviewed commentary, blogs, case studies in the public domain	Reviews of literature (although the reference list will be checked for in‐scope literature), social media

## 4 Search Strategy and Information Sources

There was a four‐step approach to searching (Peters et al. 2020; Wanyama et al. 2021). The first step was the identification of key words and index terms through initial searches and in consultation with an academic librarian. The second step was to search multiple databases: MEDLINE, CINAHL, Google Scholar and Theses Global from 1 January 2000 to 23 November 2023 (see Supporting Information S1 for a search strategy). Thirdly, the reference list of identified reports and articles was searched for additional sources. Authors of primary sources or reviews were contacted to check for additional material. The fourth step was to search for additional grey/unpublished material on websites of the World Health Organisation, the International Council of Nursing and every national nursing organisation listed on the ICN website.

## 5 Evidence Selection

Potential evidence from databases was imported into an excel spreadsheet and duplicates removed. Titles and abstracts were screened by two researchers independently against the inclusion criteria. Full texts were retrieved and imported into RefWorks. These were screened by two researchers independently against the inclusion criteria and reasons for exclusion recorded. Disagreements were resolved through discussion. In addition, we searched forward citations of included literature. Evidence from websites was reviewed by two reviewers against the inclusion criteria and reasons for exclusion recorded. The research team reviewed and agreed on the final included texts. The evidence selection was recorded using a flow diagram (Tricco et al. 2018).

## 6 Data Processing and Charting

Data from the included texts were extracted and charted to a bespoke spreadsheet (Peters et al. 2020). The spreadsheet included variables of the general characteristics of the text (citation, year, country, type of literature) as well as specific variables to answer the research question, which drew on the general WHO and ICN guidance on retention strategies (World Health Organisation 2016; Buchan et al. 2022). The variables included: the aim; the location; the types of strategy/strategies (e.g., flexible work schedules, control over work activities, attention to financial aspects of the jobs and access to other benefits, creating cohesive teams, promoting professional growth, employee recognition schemes); processes described; resources used; impact and outcome; explanations for success; explanations for failure; authors stated limitations; conclusions and implications. Quality appraisal was to be undertaken for any research studies identified appropriate tools. Only one cross‐sectional study was identified and appraised using the JBI critical appraisal tool (Moola et al. 2020).

## 7 Analysis

The charted material was used to narratively describe the breadth of the material found against the research questions for example, the range and categories of strategies, the evidence of impact or outcomes. Thereafter, thematic mapping was used, drawing on techniques from realist reviews (Peters et al. 2020) to identify from the papers any underlying explanatory mechanisms and theories for the success or otherwise of strategies. While a narrative analysis with exemplar texts was drafted and agreed with the research team.

## 8 Protocol Registration

Open Science Framework https://doi.org/10.17605/OSF.IO/TPQ56 (Drennan et al. 2023).

Ethics review was not required for the use of published material.

## 9 Results

**FIGURE 1:**
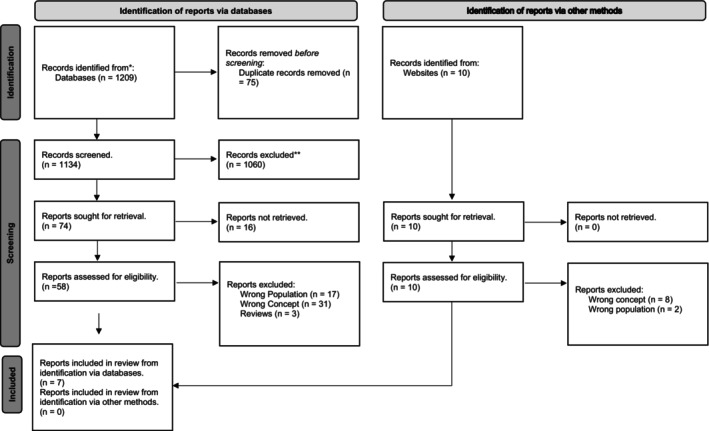
Flow diagram of results.

One thousand two hundred and nineteen records were identified, screened, and reviewed and from which seven (Cushman et al. 2001; Ellenbecker et al. 2007; Johnston et al. 2020; Linscheid and Bell 2021; Markey 2006; Pennington and Driscoll 2019; Smith‐Stoner and Markley 2007) finally met the scoping review criteria (Figure 1) (Tricco et al. 2018).

### 9.1 Characteristics

Six of the papers were from the United States (Cushman et al. 2001; Ellenbecker et al. 2007; Linscheid and Bell 2021; Markey 2006; Pennington and Driscoll 2019; Smith‐Stoner and Markley 2007) and one from the UK (Johnston et al. 2020), Table 2. Three papers reported on evidence from service managers: two from group discussions (Markey 2006; Smith‐Stoner and Markley 2007) and one from a cross‐sectional survey (Cushman et al. 2001). Three papers reported on evaluations of service initiatives to improve retention of nurses new to home visiting nursing services (Johnston et al. 2020; Linscheid and Bell 2021; Pennington and Driscoll 2019). One paper reported on a research study of employer‐reported retention strategies and their relationship with nurses' reported job satisfaction and intent to stay (Ellenbecker et al. 2007). The publication years were unevenly spread, with four published between 2001 and 2007 (Cushman et al. 2001; Ellenbecker et al. 2007; Markey 2006; Smith‐Stoner and Markley 2007) followed by over a decade of no publications, and then one publication in 2019 (Pennington and Driscoll 2019), 2020 (Johnston et al. 2020) and 2021 (Linscheid and Bell 2021).

**TABLE 2:** Characteristics of included papers.

First author, year and country	Type of paper	Aim	Methods	Participants
(Cushman et al. 2001), USA	Research	To determine home health care agency administrators' perceptions of why nurses stay in their jobs	Cross‐sectional mailed survey with questions for free text responses with qualitative thematic analysis	196 administrators responded from 44 states
(Ellenbecker et al. 2007), USA	Research	To describe the strategies implemented by home health care agencies and their effect on nurse job satisfaction and intention to leave.	Cross sectional mailed survey to home care nurses, with job satisfaction and intention to leave questionnaires, compared to results of cross‐sectional survey to administrators of the nurses' employing home care agency about retention strategies currently used.	Administrators of 123 home health care agencies in one state (New England) and 2459 nurses employed in the same home health care agencies
(Johnston et al. 2020), UK	Service innovation Evaluation	To support newly appointed nurses to a home visiting palliative care service through a 3‐month peer‐mentoring programme	Measurement of participants still in post at 12 months after the peer mentoring programme and compared to rates prior to the programme	74 registered nurses new to home palliative care services recruited over 36 months
(Linscheid and Bell 2021), USA	Service innovation evaluation	To attract and retain newly licensed RNs into home health care jobs through a 12‐month residency program with clinical preceptors	Measurement of participants still in post at 12 months after the program and compared to rates prior to the programme	28 newly licensed registered nurses appointed to home health care posts
(Markey 2006), USA	Commentary	To share successful retention strategies for home health care nurses	Teleconferenced discussion	Unknown number of chief executive officers of home health care agencies
(Pennington and Driscoll 2019), USA	Service innovation Evaluation	To improve the retention of RNs, new to home health care, through a 12‐week education programme with clinical preceptor, followed by 1 year mentorship	Measurement of participants still in post at 12 and 24 months after the program, compared to rates prior to the program	154 nurses completed the program and of those, 91 participated in the mentorship program
(Smith‐Stoner and Markley 2007), USA	Commentary	To discuss and identify successful strategies in retaining home health care nurses	A series of panel discussions in one state (Texas), enhanced with a review of literature	15 representatives of agencies with high retention levels and 512 individuals from 236 home health care agencies

### 9.2 Evidence From Administrators' Viewpoints and Evaluations

We discuss first the papers reporting senior administrators views in home visiting nursing organisations (Cushman et al. 2001; Markey 2006; Smith‐Stoner and Markley 2007), then the research paper investigating nurses' intention to stay in the context of their organisations' stated retention strategies (Ellenbecker et al. 2007), and finally the papers reporting service innovations for retaining RNs new to home visiting nursing (Johnston et al. 2020; Linscheid and Bell 2021; Pennington and Driscoll 2019).

*The views of* *home visiting* *nursing service administrative staff*. Three US papers reported the views of senior administrators (Cushman et al. 2001; Markey 2006; Smith‐Stoner and Markley 2007).

The two commentary papers described presentations by officials of home health care agencies which had high nurse retention rates, followed by round table discussions with a wider group of home health care senior administrators (Markey 2006; Smith‐Stoner and Markley 2007). Markey, a home health agency nurse executive officer and head of a professional visiting nurse organisation, reported that one of the presenting agencies had reduced a turnover rate from 30% to 5% using multiple, but unspecified, strategies (Markey 2006). From the ensuing discussions, multiple strategies were reported to be used, which included the following: training tailored to new graduates, changes to nurses' benefits packages, tuition reimbursement and flex time (Markey 2006). No outcome data or evidence of effectiveness was presented.

The second commentary paper, written by a university professor of nursing and a nurse executive of a health care quality improvement agency, provided no data from agencies with reported high nurse retention rates, but again multiple retention strategies were said to be used (Smith‐Stoner and Markley 2007). These multiple strategies discussed by presenters and attendees were the categorised by the authors (Smith‐Stoner and Markley 2007). The first category was using financial strategies for example, payment of market rate salaries, reimbursement for petrol mileage. The second category of strategies was called operational and included: orientation and clinical education programmes for nurses new to home care; flexible scheduling and early termination of low performing nurses. The third category was described as interpersonal, this included: having supervisors skilled at communication and open to feedback (Smith‐Stoner and Markley 2007).

US home health administrators' views were also reported from a cross‐sectional survey (Cushman et al. 2001). On critically appraisal of the paper, several methodological and results elements were missing (Cushman et al. 2001). The paper reported qualitative (free text) responses to one question on home health agency administrators' perceptions of successful retention strategies (Cushman et al. 2001). The question was: *what do you think are the one or two most important reasons registered nurses (RNs) stay at your agency*? (Cushman et al. 2001, 64). The paper reported on unspecified number of respondents to this question from the overall 193 respondents (of 2300) to the survey. The authors reported the most important reason reported for retention was the strategy of allowing flexibility of working hour of the nurses. Other retention reasons were reported as: the relationship the nurses had with the agency and colleagues; the competitive salary and packages of benefits; the enjoyment of the autonomy in the work and the relationship with patients (Cushman et al. 2001).

*The intentions of nurses in the context of their employing organisation' stated retention strategies*. One US research paper reported on a regression analysis of home health care nurses (*n* = 2459) self‐reported intention to stay and job satisfaction (Ellenbecker et al. 2007). This paper when critically appraised met the quality criteria for cross sectional surveys (Moola et al. 2020). The study analysed the RNs responses in the context of the nurses employing organisations (*n* = 123) self‐reported use, or not, of eight pre‐specified retention strategies (Moola et al. 2020). The most frequently reported (94%–82% of respondents) retention strategies used were: flexible work schedules, opportunity for control over work, employee recognition strategies and shared decision making. The multivariate regression analysis used the mean scores by organisation of job satisfaction and intent to stay and found that none of the eight retention strategies directly affected nurses' intention to stay in their job. The only retention strategy reported to have a statistically significant effect on job satisfaction was that of shared decision making. The authors observed in the study limitations that a different level of analysis (i.e., at an individual nurse level rather than using organisation mean averages) might have produced different results (Ellenbecker et al. 2007).

*Service innovations to retain RNs new* *to home visiti* *ng* *nursing*. Three papers described the outcomes of service improvement initiatives aimed at improving the retention rate of RNs new to home visiting nursing services (Johnston et al. 2020; Linscheid and Bell 2021; Pennington and Driscoll 2019). Each paper described how the innovation was a response to high leaving rates in RNs new to working in patients' homes, often within a few months of recruitment to post. Each paper reported retention rates of RNs following the innovation, but none considered other contextual or confounding factors in the analysis.

Two of the innovations were for experienced nurses new to home visiting nursing (Johnston et al. 2020; Pennington and Driscoll 2019) and one was for newly qualified nurses also new to home visiting nursing (Linscheid and Bell 2021). The innovations were: the provision of peer mentoring for 3 months (Johnston et al. 2020); a 12‐week knowledge and clinical skills programme with a workplace preceptor orientation (Pennington and Driscoll 2019); and a combined orientation with clinical skills and workplace preceptor plus 12 months seminar programme (Linscheid and Bell 2021). Reductions in experienced RN leaving rates at 12 months were reported when compared to the previous year (Johnston et al. 2020; Linscheid and Bell 2021). Retention rates were reported as high at 24 months for the newly qualified nurses, but no comparative figures were given (Pennington and Driscoll 2019).

### 9.3 Synthesis of Findings

Multiple strategies were reported to be used but not detailed nor evidence of impact on retentions rates found. We analysed all included papers to identity the frequency of types of strategy and categorise them by underlying mechanism (Table 3). We identified eight underlying mechanisms: (1) finance incentives; (2) work schedule flexibility; (3) team level management; (4) positive feedback on job performance; (5) team level interpersonal relationships; (6) the work organisation and resources; (7) support to individual's development in knowledge, clinical skill and confidence and (8) participation in organisation's decision making. All four papers of administrators' reported retention strategies were based on underlying mechanisms of finance incentives and work schedule flexibility to meet individual RN requirements (Cushman et al. 2001; Ellenbecker et al. 2007; Markey 2006; Smith‐Stoner and Markley 2007). The remaining six mechanisms were not found in all papers, possibly because of the brevity of the reported questions in one paper (Cushman et al. 2001), the brevity in reporting in another (Markey 2006) and the brevity of description of retention strategies in a third paper (Ellenbecker et al. 2007). However, the absence of some of the types of strategies in all papers might also reflect their actual absence. Ellenbecker et al. found that while multiple retention strategies were reported to be used, not all strategies were used by all employers (Johnston et al. 2020). None of these papers provided quantitative evidence of any impact on RN actual retention rates.

**TABLE 3:** Synthesis of reported retention strategies and underlying mechanism.

Type of mechanism underlying strategy	Reported retention strategy	(Cushman et al. 2001)	(Ellenbecker et al. 2007)	(Markey 2006)	(Smith‐Stoner and Markley 2007)	(Johnston et al. 2020)	(Linscheid and Bell 2021)	(Pennington and Driscoll 2019)
Financial incentives	Offer competitive salaries	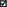	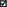		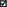			
	Benefits packages	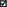		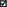				
	Reimbursement for petrol mileage				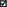			
	Small monetary rewards in recognition of quality service				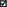			
	Payment for continuing education fees			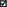	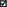			
Flexible work hours for individual RNs	Flexible work schedules	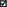	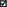	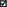	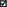			
Team level management	Supervisors skilled at communication and open to feedback				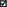			
	Frequent contact by supervisors with nurses and patients				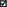			
	Opportunity for control over work	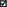	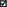					
	Early termination of low performing nurses				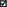			
Positive feedback on job performance	Employee recognition strategies		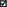		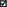			
	Relationships with patients	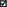						
Support to individual's development in knowledge, clinical skill and confidence	Opportunities for professional growth		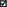					
	Training tailored to new graduates and RNs new to home nursing			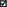	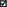	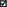	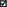	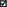
Team level interpersonal relationships	Fostering of a professional, respectful culture in the team and agency	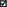			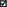			
	Relationships and camaraderie with colleagues	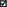			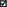	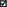	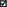	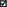
Work organisation and resources	The use of case manager models of providing care which included office time for adequate management of patient care and documentation				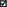			
	High ratios of RNs to patients thus reducing supervising activities for non‐professionally qualified assistants				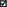			
	Agency reduced job demands		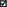					
	Enhanced workplace safety		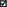					
Participation in organisation's decision making	Shared decision making		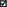					
	Nurse involvement in decisions affecting clinical practice				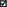			

Targeted interventions to retain RNs new to home visiting nursing were reported successful in five papers (Johnston et al. 2020; Linscheid and Bell 2021; Markey 2006; Pennington and Driscoll 2019; Smith‐Stoner and Markley 2007). The evaluation papers stated these interventions were addressing the lone working nature of home visiting nursing and the feelings of isolation reported by many new to this type of nursing (Johnston et al. 2020; Linscheid and Bell 2021; Pennington and Driscoll 2019). Our analysis also suggested the underlying mechanism was to provide support to individual RN's development in knowledge, clinical skills, and confidence in nursing in patients homes (Table 3).

## 10 Discussion

This scoping review identified only seven papers published internationally in the past 24 years which provided any evidence of the effectiveness, or otherwise, of retention strategies for nurses in home visiting nursing services (Cushman et al. 2001; Ellenbecker et al. 2007; Johnston et al. 2020; Linscheid and Bell 2021; Markey 2006; Pennington and Driscoll 2019; Smith‐Stoner and Markley 2007). This is a very small number of papers. It is perhaps even more surprising to find so little attention having been paid to retaining nurses in these home visiting services over a period when internationally health care systems aimed to support more older adults to age in place and reduce demands within the high‐cost hospital sector (World Health Organization 2002). Explanations for this seeming lack of attention could be that: that home visiting nursing services are not universally provided in all countries or that these types of services are simply overshadowed by the global shortages of RNs to work in the hospital sector (Buchan et al. 2022).

To our knowledge this is the first review to investigate the evidence of effective strategies in retaining nurses in home visiting services. We found three papers reporting administrators' views of effective strategies (Cushman et al. 2001; Markey 2006; Smith‐Stoner and Markley 2007) and one research paper investigating RNs reported intention to leave their jobs in the context of their employing agencies reported recruitment strategies (Ellenbecker et al. 2007). All papers reported organisations were using multiple strategies but not the detail or the combinations of strategies. Strategies that addressed financial rewards and flexibility in hours were reported as effective in all papers. None of these papers provided evidence of the impact of the strategies on retention rates. We have identified an evidence gap. Further investigation is needed into the relationships between different combinations of strategies and actual retention rates of RNs in home visiting services. We suggest that any research should be comparative and include a wide variety of socio‐economic contexts, as it is well known that certain types of areas such as rural areas have greater difficulties in retaining staff (World Health Organisation 2016). We further recommend that different methodologies are used for evaluations of service interventions for example, longitudinal studies across multiple organisations. We suggest these different methodologies would provide more robust evidence for clinical managers and policy makers.

We found evidence for targeted intervention strategies to support RNs new to home visiting nursing in five of the seven included papers. Each included paper had different types of components and time periods for their programmes. A review of these type of programmes for newly qualified nurses in hospital settings also noted great variation in the components (Brook et al. 2019). Further research is required to help specify the most effective components in the home visiting nursing setting.

It was surprising to find such a concentration of papers (6 of 7) from the United States, given other countries in Europe, Australasia and Southeast Asia have long histories of home visiting nursing services (Drennan 2019). It might be argued that given the variations in the way these services are organised in different countries, the evidence found in the review has limited value. We suggest that our identification of underlying mechanisms provides a route to theoretical generalisation which can be tested in further research. We identified eight mechanisms underlying retention strategies reported in the seven papers. These were: (1) finance incentives; (2) work schedule flexibility for individuals; (3) team level management; (4) positive feedback on job performance; (5) team level interpersonal relationships; (6) the work organisation and resources; (7) support to individual's development in knowledge, clinical skill and confidence and (8) participation in organisation's decision making. These mechanisms map onto many of the extrinsic and intrinsic factors identified in theoretical frameworks of factors influencing nurses to remain or leave their jobs (Gilmartin 2013; Ellenbecker 2004). Our analysis of underlying mechanisms relevant to home visiting nursing services is novel and requires further investigation and validation. However, we suggest that the identified mechanisms may have utility in directing team leaders and managers in home visiting nursing services as to where to prioritise retention efforts. Empirical evidence is required to answer questions such as whether home visiting nursing services with retention strategies addressing greater numbers of mechanisms also report greater retention of nurses.

### 10.1 Strengths and Limitations

This review has both limitations and strengths. A strength was that we followed international guidance and tools in the conduct of the review. We published the protocol for the review prior to commencement to ensure transparency and credibility. We have reported the review following international guidance to ensure transparency in our methods and validity to our findings and interpretation. A limitation was that we only included English language papers and may therefore have excluded relevant papers published in languages other than English, for example, from Japan which has a long history of home visiting nurses. However, home visiting nurses from Nordic countries and Japan regularly publish in English language academic publications. We sought relevant grey literature internationally from websites of nursing associations listed on the ICN website. However again, we may have omitted relevant publications through exclusion of languages other than English. The review is further limited by the small number of papers, the majority published from one country and therefore generalisability could be questioned. A strength of the review is that we have offered an analytical framework of underpinning mechanisms which are generalisable across health systems. We have argued that this requires further testing and investigation.

## 11 Conclusions

This scoping review, investigating successful nurse retention strategies, identified a small amount of qualitative evidence and opinion on the value of multiple strategies. However, aside from the promising evidence of clinical and peer support programmes for those new to home visiting nursing services, there is little to guide clinical leaders of home visiting nursing services as to where best to focus their efforts. Our identification of eight underlying mechanisms at organisational and team level requires further validation and investigation. The lack of evidence is a pressing issue because many health care systems are looking to increase the provision of acute, long‐term, palliative and end of life care in patients' own homes. Without an expanded and stable nursing workforce delivering services into people's homes, such shifts in the provision of care are unlikely to succeed.

## Author Contributions

V.M.D., J.C.F., C.T., M.H., L.W. and E.A. have made substantial contributions to conception and design, or acquisition of data, or analysis and interpretation of data. V.M.D., J.C.F., C.T., M.H., L.W. and E.A. were involved in drafting the manuscript or revising it critically for important intellectual content. V.M.D., J.C.F., C.T., M.H., L.W. and E.A. have given final approval of the version to be published. Each author should have participated sufficiently in the work to take public responsibility for appropriate portions of the content. V.M.D. agreed to be accountable for all aspects of the work in ensuring that questions related to the accuracy or integrity of any part of the work are appropriately investigated and resolved.

## Ethics Statement

As a review of publicly available material, research ethics review was not required.

## Conflicts of Interest

The authors declare no conflicts of interest.

## Supporting information


Data S1.

## Data Availability

The data that support the findings of this study are available from the corresponding author upon reasonable request.
