# Supplementary material for: Successful Retention Strategies for Nurses in Home Visiting Nursing Services: A Scoping Review
Source: J Adv Nurs. 2025 Jun 16;82(4):2539–50. doi: 10.1111/jan.70020 (PMC12994657; doi:10.1111/jan.70020)
Supplement: Supplementary file 1 — Data S1. [file JAN-82-2539-s001.docx]

Supplementary paper 1

Medline search strategy

Database: MEDLINE

Date: 29/11/2023 Search terms

S1 community nurse OR district nurse OR home health nurse OR home visiting nurse

S2 (MM "Nurses, Community Health") OR (MM "Home

Health Nursing") OR (MM "Home Nursing+") OR (MM

"Community Health Nursing+") OR (MM "Nurses, Public Health")

S3 S1 OR S2

S4 Personnel retention OR career planning OR personnel turnover OR personnel shortage OR staff retention OR employee retention OR retaining

S5

S3 AND S4
